# Air Quality Prediction Using the Fractional Gradient-Based Recurrent Neural Network

**DOI:** 10.1155/2022/9755422

**Published:** 2022-12-09

**Authors:** Sugandha Arora, Narinderjit Singh Sawaran Singh, Divyanshu Singh, Rishi Rakesh Shrivastava, Trilok Mathur, Kamlesh Tiwari, Shivi Agarwal

**Affiliations:** ^1^Birla Institute of Technology and Science Pilani, Pilani, Rajasthan, India; ^2^Faculty of Data Science and Information Technology, INTI International University, Persiaran Perdana BBN, Putra Nilai, 71800, Nilai, Negeri Sembilan, Malaysia

## Abstract

In this study, the air quality index (AQI) of Indian cities of different tiers is predicted by using the vanilla recurrent neural network (RNN). AQI is used to measure the air quality of any region which is calculated on the basis of the concentration of ground-level ozone, particle pollution, carbon monoxide, and sulphur dioxide in air. Thus, the present air quality of an area is dependent on current weather conditions, vehicle traffic in that area, or anything that increases air pollution. Also, the current air quality is dependent on the climate conditions and industrialization in that area. Thus, the AQI is history-dependent. To capture this dependency, the memory property of fractional derivatives is exploited in this algorithm and the fractional gradient descent algorithm involving Caputo's derivative has been used in the backpropagation algorithm for training of the RNN. Due to the availability of a large amount of data and high computation support, deep neural networks are capable of giving state-of-the-art results in the time series prediction. But, in this study, the basic vanilla RNN has been chosen to check the effectiveness of fractional derivatives. The AQI and gases affecting AQI prediction results for different cities show that the proposed algorithm leads to higher accuracy. It has been observed that the results of the vanilla RNN with fractional derivatives are comparable to long short-term memory (LSTM).

## 1. Introduction

With the increase in urbanization, industrialization, and traffic in the cities, the air pollutants are increasing and air quality is reducing [1]. To keep a check on the extent of air pollution, the US Environment Protection Agency has introduced a parameter called the air quality index (AQI) which tracks the daily effects of air pollutants [2]. AQI is a numerical value between 0 and 500; when the value of AQI is 0, the air quality is adequate, and if the value of AQI is 500, then the air quality is hazardous. AQI is calculated by considering major air pollutants, such as carbon monoxide (CO), nitrogen dioxide (NO_2_), ozone (O_3_), particulate matter (PM_10_ and PM_2.5_), and sulphur dioxide (SO_2_). These pollutants are the residual gases and particles emitted from vehicles, industries, and due to climate change [3].

Biomass and coal-burning highly increase the levels of particulate matter (PM_10_ and PM_2.5_) that causes haze in the air. These particles deteriorate the air composition and cause respiratory problems in living beings. Moreover, haze reduces the visibility that further affects the economic sectors such as tourism and agriculture [4]. The combustion of fossil fuels is carried out in several industries which are the main contributors of SO_2_ and NO_2_ in air [5, 6]. The motorized vehicles and combustion of fossil fuels also emit CO, which is another major pollutant responsible for worsening the air quality. CO is highly poisonous and can even lead to mortality on long exposure [7]. Another major pollutant is ground-level ozone O_3_, obtained from the combination of two primary pollutants, nitrogen oxides (NO_*x*_) and volatile organic compounds (VOCs). The 95% of these primary pollutants come from oil, coal, and gasoline combustion in vehicles, industries, power plants, and households, upstream gas and oil production, combustion of residual woods, and the evaporated liquid fuels [8]. Exposure to ozone can significantly affect human health, cause asthma, and can lead to premature mortality [9]. In addition, ozone can adversely affect vegetation, damage flowers and shrubs, and reduce crop productivity [10, 11].

Air pollution is not a local phenomenon; the current quality of air is dependent on its history. The industrialization has massively impacted the environment, especially the air quality [12]. The levels of air pollutants like ground-level ozone and particulate matter are also getting influenced by modifying weather patterns that occurred due to climate change [13, 14]. The change in climate affects the temperature, humidity levels, and wind patterns, which in turn influences the air quality. In addition, the naturally occurring emissions, for example, wind-blown dust and wildfires, get provoked by climate-driven changes in meteorology that affect the air quality. The uncontrolled emission of air pollutants is gradually causing air pollution. Continuous exposure to polluted air is severely affecting human health [15] and leading to the development of lung, heart, and skin diseases [16]. Six of the world's top 10 most polluted cities are from India. Air pollution has been observed as the second biggest risk factor which is causing diseases in India and thus affecting its economy. Thus, there is a need to keep a check on air pollution in Indian cities. Each city has its unique features such as population per square km, temperature, humidity level, climate, vehicles, and industries in the region, and thus, it is better to study air pollution region-wise. Generally, the air quality in tier I and urban cities is low and it is required to give more attention in such areas.

To prevent the serious consequences of air pollution, several forecasting techniques for AQI are being developed. Based on target objectives, the techniques and approaches of forecasting are being expanded and improved. Traditional AQI forecasting techniques involve statistical techniques such as autoregressive integrated moving average (ARIMA) [17, 18], principal component regression (PCR) [19], multiple linear regression (MLR) [20], and grey models [21, 22]. These models perform well, but with the high increase in pollution, more accurate methods are required. These models are linear and thus are unable to capture the nonlinear traits [23, 24]. Even with a large amount of data, not much increase is seen in the accuracy of these models. The performances of the statistical techniques have been improved by developing hybrid techniques [25]. Artificial intelligence-based techniques are capable of analyzing the nonlinear data and thus are being recently used in the time series forecasting [26, 27]. With the availability of sufficient amount of data and computational support, AQI forecasting is being done with a deep neural network [28]. But these methods require to learn large number of parameters. Thus, a simpler and accurate method has been developed in this study using a vanilla RNN. The current level of air pollution in any area is also dependent on AQI status in the past. To capture the history dependency, fractional derivatives have been employed in the back propagation algorithm to train the vanilla RNN for the prediction of AQI in Indian cities.

**Figure 1:**
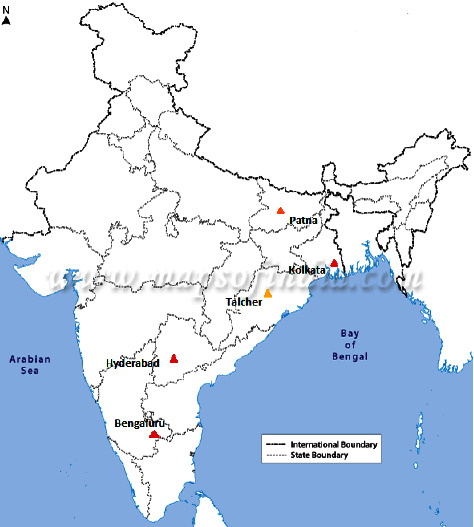
Map of India showing the locations of tier (I), tier II, and tier III cities represented by red, orange, and yellow color, respectively.

In this study, five cities of different tiers are considered for AQI prediction. Bengaluru, Kolkata, and Hyderabad are tier I cities, while Patna and Talcher are tier II and tier III cities, respectively, as shown in Figure 1. The major air pollutants of Kolkata are also predicted using the proposed approach. The results show that the proposed method achieves minimum error on some fractional orders. Also, the obtained results are comparable to the LSTM. The rest of the study is structured as follows: Section 2 briefly explains the related work. The proposed approach is presented in Section 4.  Section 5 discusses the experimental results obtained, followed by conclusion and future scopes in Section 6 and Section 7, respectively.

## 2. Preliminaries

Factional calculus is a 300-year-old branch of mathematics that deals with derivatives and integrals of noninteger order, i.e., order can be any number, be complex or real. Earlier, this domain was only theoretical involving rigorous calculations, but these derivatives are used in practical applications as well [29, 30]. Several versions of fractional derivatives and integrals have been introduced till now, where each version has unique characteristics. The most widely used versions are described.

(i) **Riemann–Liouville (RL) fractional integral operator: **This is the most frequently used version of fractional integral [31]. The *α* order RL fractional integral is expressed as follows:
  (1) $$\begin{array}{c} {}_{a}D_{t}^{-\alpha }f\left(t\right)=\frac{1}{\Gamma \left(\alpha \right)}\int_{a}^{t}{\left(t-\eta \right)}^{\alpha -1}f\left(\eta \right)d\eta \text{.} \end{array}$$
Here, *α* > 0, *t* > *a*, *t*, *a* ∈ *ℝ*, the function Γ(·) is the Gamma function, and *f* is a piecewise continuous function on [0, *∞*) and integrable on any finite subinterval of (0, *∞*).
(ii) **Riemann–Liouville (RL) fractional derivative:** This is the natural generalization of integer-order derivative, as this fractional derivative version and ordinary derivative are left inverse of integrals, i.e., _*a*_**D**_*t*_^*α*^*D*^−*α*^*f*(*t*) = *D*^0^*f*(*t*) = *f*(*t*). For *t* > *a*, *α* > 0, and *n* ∈ *ℕ*, such that *n* − 1 < *α* < *n*; the derivative of order *α* is evaluated by differentiating the *n* − *ν* order RL integral of function *f*(*t*)*n* times,
  (2) $$\begin{array}{c} {}_{a}D_{t}^{\alpha }f\left(t\right)=D^{n}\left[{}_{a}D_{t}^{-\left(n-\alpha \right)}f\left(t\right)\right]\text{,} \end{array}$$
i.e.,
  (3) $$\begin{array}{c} {}_{a}D_{t}^{\alpha }f\left(t\right)=\left\{\begin{array}{cc} \frac{1}{\Gamma \left(n-\alpha \right)}\frac{d^{n}}{dt^{n}}\overset{t}{\underset{a}{\int }}\frac{f\left(\eta \right)}{{\left(t-\eta \right)}^{\nu +1-n}}\;d\eta \text{,} & n-1<\alpha <n\text{,}\\ \frac{d^{n}}{dt^{n}}f\left(t\right)\text{,} & \alpha =n\text{.} \end{array}\right. \end{array}$$
It is the *α* order RL fractional derivative [31]. But this definition has some disadvantages as well. The most significant disadvantage is that RL derivative of order *α*, (<1) of a constant is not zero.
(iii) **Caputo's fractional derivative:** For*t* > *a*, *α* > 0, and *n* ∈ *ℕ*, such that *n* − 1 < *α* < *n*, the Caputo's derivative of order *α* is obtained by evaluating the *n* − *α* order RL integral of *n*^th^ order derivative of function *f*(*t*), i.e., _*a*_^*C*^*D*_*t*_^*α*^*f*(*t*)=_*a*_*D*_*t*_^−(*n* − *α*)^[*D*^*n*^*f*(*t*)], that gives the following:
  (4) DtαaCft=1Γn−α∫atfnηt−ηα+1−n dη,n−1<α<n,dndtnft,α=n.
It is called the *α* order Caputo's fractional derivative. Caputo gave this definition of fractional derivative in 1967 for overcoming the limitations of RL derivative [32]. The Caputo's derivative of order *α* > 0, *n* − 1 ≤ *α* < *n* for a constant *c* is zero. This version increased the applicability of fractional derivatives in modelling real world problems. Thus, in this study, Caputo's version of fractional derivatives has been used.
(iv) **Grünwald–Letnikov (GL) fractional derivative:** The limit definition of fractional derivative was given by Anton Karl Grünwald and Aleksey Vasilievich Letnikov in 1867 and 1868, respectively [31]. Without any assumptions on differentiability of the function for *α* > 0, the *α*-order GL derivative of function *f* is expressed as follows:
  (5) $$\begin{array}{c} {}_{a}D_{t}^{\alpha }f\left(t\right)=\lim_{\begin{array}{c} h⟶0\\ nh=t-a \end{array}}h^{-\alpha }\overset{n}{\underset{r=0}{\sum }}{\left(-1\right)}^{r}\left(\begin{array}{c} \alpha \\ r \end{array}\right)f\left(t-rh\right)\text{.} \end{array}$$
Here, *h* is the step size and $\left(\begin{array}{c} \alpha \\ r \end{array}\right)=\Gamma \left(\alpha +1\right)/\Gamma \left(\alpha -r+1\right).\Gamma \left(r+1\right)$ with the Gamma function Γ(·).

Clearly, the limit definition of first-order derivative shows that evaluation of derivative involves usage of only two points. But it can be seen from limit definition of fractional derivatives and from the Caputo's definition in equation (4) and equation (5), respectively, that their evaluation involves usage of value of the function at all past points. This makes the fractional derivatives to be a nonlocal operator and incorporate memory to the systems. Due to the memory property and availability of software and other tools, fractional calculus has been used in numerous applications of science and engineering. These have been widely used in viscoelasticity [33], biology [34], signal and image processing [35], stock market [27, 36], economics [37], and in other domains with history dependency [38]. Moreover, the order of differentiation acts as a degree of freedom in the optimization process.

The nonlocality of fractional derivatives has been the major motivation for their application in different domains. Fractional calculus has been successfully used for air quality prediction [39–41]. Fractional derivative-based Kalman filter has been introduced to measure the pollutant emission and hence the air quality [39]. Several variants of fractional Kalman filters have been developed using different fractional-order derivatives version for improving the prediction accuracy [40–42]. In these air-quality models, fractional calculus is incorporated because of its long-term memory and nonlocal nature. Fractional calculus has been successfully applied in the training of artificial neural networks [43–46]. After replacing integer-order derivative by fractional-order derivative in the back propagation of the training algorithm, the update rule gets updated as follows:

(6) $$\begin{array}{c} \Delta w=-\eta \frac{\delta^{\alpha }E}{\delta w^{\alpha }}\text{,} \end{array}$$

where *η* and *α* are the learning rate and fractional order of differentiation, respectively. Chen [47] employed the fractional derivative in their backpropagation method for feed-forward neural networks (FNNs) in 2013. The simulation results showed that fractional derivative-based FNNs had a substantially better convergence speed than integer-order FNNs. The fractional derivatives have been successfully applied in the backpropagation learning algorithm of the radial basis function network [48], recurrent neural network [46], convolutional neural network [49], and even in deep neural networks and have shown significant improvement in accuracy. In our study, the effect of using fractional derivatives in the learning of the neural network has been analyzed for the prediction of air quality in few Indian cities. Earlier too, the effectiveness of fractional derivatives has been shown for nonlinear system identification, pattern classification, and Mackey–Glass chaotic time series prediction [46].

## 3. Fractional Gradient-Based Backward Propagation Algorithm

In this section, we introduce the fractional-order truncated backpropagation through the time algorithm on the RNN with 10 neurons in a layer. This backpropagation algorithm considers the truncated depth of the input data and the state of the network, which makes the algorithm computationally efficient. For the implementation of the backpropagation algorithm, the mean squared error at an instant is considered as follows:

(7) $$\begin{array}{c} E\left(s\right)=\frac{1}{2}\underset{i\in \Omega }{\sum }{\left(\Phi \left(u_{i}\left(s\right)\right)-x_{i}\left(s\right)\right)}^{2}=\frac{1}{2}\underset{i\in \Omega }{\sum }{\left(e_{i}\left(s\right)\right)}^{2}\text{,} \end{array}$$

where *i* is the output neuron, Φ(*u*_*i*_(*s*)) and *x*_*i*_(*s*) are the actual output and the expected output of the *i*_*th*_ neuron at time *s*, and *u*_*i*_(*s*) = ∑_*j*∈Ω_*w*_*ij*_*v*_*j*_(*s*) at time *s*, where *w*_*ij*_(*s*) is the weight of a signal from *j*_*th*_ neuron to *i*_*th*_ and *v*_*j*_(*s*) is the output of *j*_th_ neuron at time *s*; then, the update rule becomes as follows:

(8) $$\begin{array}{c} w_{ij}\left(s+1\right)=w_{ij}\left(s\right)-\eta \nabla_{w_{ij}}^{\alpha }E\left(s\right)\text{,} \end{array}$$

where *η* is the learning rate and ∇_*w*_*ij*__^*α*^ represents the factional gradient w.r.t *w*_*ij*_. Now, ∇_*w*_*ij*__^*α*^*E*(*s*) can be evaluated by applying the approximated chain to the error function. The actual chain rule applicable on fractional derivatives is complicated and involves special mathematical functions; thus, several approximated chain rules have been developed for fractional derivatives. [50–53] The chain rule given by expression (14) has been obtained by using fractional Taylor's series expansion for differentiable function. Consider a differentiable function, say *f* then for a small *h*,

(9) $$\begin{array}{c} f\left(x+h\right)=f\left(x\right)+\frac{h^{\alpha }}{\Gamma \left(1+\alpha \right)}f^{\left(\alpha \right)}\left(x\right)+\frac{h^{2\alpha }}{\Gamma \left(2+\alpha \right)}f^{\left(2\alpha \right)}\left(x\right)+\frac{h^{3\alpha }}{\Gamma \left(3+\alpha \right)}f^{\left(3\alpha \right)}\left(x\right)+\cdots \text{.} \end{array}$$

Then,

(10) $$\begin{array}{c} \frac{\Delta f\left(x\right)}{h^{\alpha }}=\frac{1}{\Gamma \left(1+\alpha \right)}f^{\left(\alpha \right)}\left(x\right)+\frac{h^{\alpha }}{\Gamma \left(2+\alpha \right)}f^{\left(2\alpha \right)}\left(x\right)+\frac{h^{2\alpha }}{\Gamma \left(3+\alpha \right)}f^{\left(3\alpha \right)}\left(x\right)+\cdots \text{.} \end{array}$$

Taking limit *h*⟶0, we get

(11) $$\begin{array}{c} \lim_{h⟶0}\frac{\Delta f\left(x\right)}{h^{\alpha }}=\frac{1}{\Gamma \left(1+\alpha \right)}f^{\left(\alpha \right)}\left(x\right)\text{.} \end{array}$$

Also, lim_*h*⟶0_Δ^*α*^*f*(*x*)/*h*^*α*^=*f*^(*α*)^(*x*),

(12) $$\begin{array}{c} \Rightarrow \Delta^{\alpha }f\left(x\right)\approx \Gamma \left(1+\alpha \right)\Delta f\left(x\right)0<\alpha <1\text{.} \end{array}$$

From above equation, we can also say *d*^*α*^*f*(*x*) ≈ Γ(1 + *α*)d*f*(*x*)0 < *α* < 1. Using this result,

(13) $$\begin{array}{c} \frac{d^{\alpha }f\left(v\left(x\right)\right)}{dx^{\alpha }}=\frac{d^{\alpha }f\left(u\left(x\right)\right)}{d^{\alpha }v}\frac{d^{\alpha }v}{dx^{\alpha }}=\frac{\Gamma \left(\alpha +1\right)df\left(v\left(x\right)\right)}{\Gamma \left(\alpha +1\right)dv}v_{x}^{\left(\alpha \right)}\left(x\right)=g_{u}^{\prime }\left(v\right).v_{x}^{\left(\alpha \right)}\text{.} \end{array}$$

Hence,

(14) $$\begin{array}{c} D_{x}^{\alpha }f\left(v\left(x\right)\right)=D_{u}^{\prime }f\left(v\right).D_{x}^{\alpha }v\left(x\right)\text{.} \end{array}$$

After using the abovementioned fractional chain rule, we get

(15) $$\begin{array}{c} \nabla_{w_{ji}}^{\alpha }E\left(s\right)=\frac{\partial E\left(s\right)}{\partial e_{j}\left(s\right)}\cdot \frac{\partial e_{j}\left(s\right)}{\partial v_{j}\left(s\right)}\cdot \frac{\partial v_{j}\left(s\right)}{\partial u_{i}\left(s\right)}\cdot \frac{\partial^{\alpha }u_{i}\left(s\right)}{\partial w_{ji}^{\alpha }\left(s\right)}\text{.} \end{array}$$

Suppose *∂E*(*s*)/*∂u*_*j*_(*s*)=*δ*_*j*_(*s*), then

(16) $$\begin{array}{c} \frac{\partial E\left(s\right)}{\partial e_{j}\left(s\right)}\cdot \frac{\partial e_{j}\left(s\right)}{\partial v_{j}\left(s\right)}\cdot \frac{\partial v_{j}\left(s\right)}{\partial u_{i}\left(s\right)}=\frac{\partial E\left(s\right)}{\partial u_{j}\left(s\right)}\cdot \frac{\partial^{\alpha }u_{i}\left(s\right)}{\partial w_{ji}^{\alpha }\left(s\right)}=\delta_{j}\left(s\right)\cdot \frac{\partial^{\alpha }u_{i}\left(s\right)}{\partial w_{ji}^{\alpha }\left(s\right)}\text{.} \end{array}$$

Now, as in this study Caputo's version of fractional derivative is being used and _0_^*C*^*D*_*x*_^*α*^*x*^*p*^ = Γ(*p* + 1)*x*^*p*−*α*^/Γ(*p* − *α* + 1), for *p* > −1, then the following holds:

(17) $$\begin{array}{c} \frac{\partial^{\alpha }u_{i}\left(s\right)}{\partial w_{ji}^{\alpha }\left(s\right)}=\frac{w_{ji}^{1-\alpha }\left(s\right)v_{i}\left(s\right)}{\Gamma \left(2-\alpha \right)}\text{.} \end{array}$$

Thus, from equations (4), (16), and (17), the following final update rule is obtained:

(18) $$\begin{array}{c} w_{kj}\left(s+1\right)=w_{kj}\left(s\right)-\eta \overset{n}{\underset{s=n-h+1}{\sum }}\delta_{k}\left(s\right)\frac{w_{kj}^{1-\alpha }\left(s\right)v_{j}\left(s\right)}{\Gamma \left(2-\alpha \right)}. \end{array}$$

## 4. Proposed Approach

In this study, the vanilla RNN has been employed to predict the AQI value of a day based on the previous sequential AQI data of five different cities. RNNs are capable of learning the sequential pattern of historical data. Furthermore, the accuracy of the system has been improved by incorporating memory into the system using the fractional gradient descent algorithm.

### 4.1. Data Exploration and Processing

In this study, the continuous AQI data have been used to predict future unseen AQI values. For each city, a continuous-time patch of around 1000 data points has been used from the AQI dataset. The sample data can be seen in Table 1. For constructing the training data, a min-max scaler has been used to scale the data values between 0 and 1. The predicted values are also obtained between 0 and 1, which are inverse-transformed later to evaluate the final predicted AQI value.

### 4.2. Neural Network Architecture and Training

**Figure 2:**
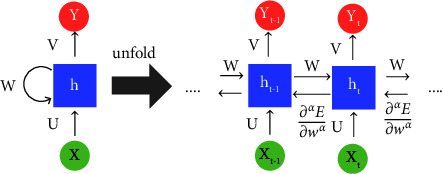
RNN architecture with fractional backpropagation.

The vanilla RNN has been used in the proposed model which has a single-layered architecture with 10 nodes in it. The fractional gradient-based RNN model is built from scratch using the NumPy library, and the Pandas library is used for data preprocessing. Forward propagation and fractional gradient-based back propagation as given by (18) have been used for training the vanilla RNN. On the other hand, for the LSTM, the TensorFlow library is used to produce all the results, and the integer-order gradient descent algorithm is used to train the model. Backpropagation has been used in both the models through 10 days (timestamps) which would predict the AQI data for the next (11^*th*^) day. Training and testing sets have 600–800 and 100 data values, respectively. For the initializing of weights for the models, Xavier's initialization has been used with 0.1 learning rate; 80 epochs were used to train the model for each city and each fractional order.  Figure 2 shows the architecture of the RNN with fractional gradient-based backpropagation.

### 4.3. Evaluation Parameter

The standard evaluation metrics for forecasting models *viz* root mean squared error (RMSE) and mean absolute percentage error (MAPE) have been employed to assess the performance of the proposed model in the prediction of AQI of different Indian cities and the major pollutants in one of those cities. The lesser the value of RMSE and MAPE, the better the performance of the predictor. These errors measure the performance of forecasting, climatology, and regression analysis for verifying the experimental results. The detailed information related to these parameters is provided.

The root mean square error (RMSE) is the square root of the average of the squared difference between the actual and predicted value. RMSE can be expressed by the following expression:

(19) $$\begin{array}{c} RMSE=\sqrt{\frac{\sum_{t=1}^{N}{\left(\hat{o_{t}}-o_{t}\right)}^{2}}{N}}\text{,} \end{array}$$

where $\hat{o_{t}}$ is the predicted value and *o*_*t*_ is the expected output for iteration *t*, which are observed for *N* times.

The mean absolute percentage error (MAPE) is the average percentage of the absolute difference between the actual and predicted values divided by the actual value for each time period [54]. MAPE can be expressed by the following expression:

(20) $$\begin{array}{c} \text{MAPE}=\frac{100}{N}\overset{N}{\underset{t=1}{\sum }}\frac{\left|\hat{o_{t}}-o_{t}\right|}{\left|o_{t}\right|}, \end{array}$$

where $\hat{o_{t}}$ is the predicted value and *o*_*t*_ is the expected output for iteration *t*, which are observed for *N* times. This is a form of percentage error, which has helped in the analysis of the proposed model in different situations.

## 5. Results and Analysis

This section describes the AQI dataset of five cities chosen, the results obtained by using the proposed approach on the AQI data, and the discussion of comparison between predictions of LSTM and the proposed approach on different fractional orders. The performance of networks is measured using RMSE and MAPE.

### 5.1. Dataset

The AQI dataset of five cities for 2015–2020 is considered which is publicly available at the official portal of the Central Pollution Control Board, Government of India (http://cpcb.nic.in/). The dataset consists of daily air quality levels at various stations across multiple cities in India which are obtained by averaging out the hourly value of AQI. Indian cities chosen for the analysis includes Kolkata, Hyderabad, Bengaluru, Patna, and Talcher. Basic information related to these cities is as follows:

- **Kolkata** (22°34′03^″^N 88°43′57^″^ E), located in West Bengal, is a tier I and the seventh most populous city of India with third-most populous metropolitan area. The concentration of pollutants such as sulphur dioxide and nitrogen dioxide remains within the limit, but the presence of particulate matter in air is high and is increasing over the years. Due to this, air pollution is severe and is causing respiratory ailments such as lung cancer.
- **Bengaluru** (12°58′44^″^ N 77°35′30^″^ E), located in Karnataka, is also a tier I and the third most populous city of India with fifth most populous metropolitan area. Bengaluru is also considered as “Silicon Valley of India” because it is the nation's top IT exporter. This IT hub region is the most polluted and is causing several environmental issues. Due to the large population, Bengaluru generates tonnes of solid waste which is polluting the environment. Thus, the large population and IT hub of Bengaluru is the major reason for air pollution.
- **Hyderabad** (17°21′42^″^ N 78°28′29^″^ E), located in Telangana, is also a tier I and the fourth most populous city of India with sixth most populous metropolitan area. Again, due to the large population, increased economic activity, and rapid urbanization, tonnes of solid waste are generated, and disposal of such waste becomes hazardous and pollutes the environment. The particulate matter (*PM*_10_) dispersed in the atmosphere causes around 2500 deaths each year.
- **Patna** (25°36′0^″^ N 85°6′0^″^ E), located in Bihar, is a tier II city with a high population. Air pollution is a major issue in this city. The situation in winter becomes even worse due to dense smog, leading to an increase in mortality. Patna was declared as the second most air polluted city in India, in the WHO survey of 2014.
- **Talcher** (20°57′0^″^ N 85°13′48^″^ E), located in Angul district of Orissa, is a tier III city. This is a small city with less population, but Talcher has the country's biggest coalfield with the highest coal reserve of around 52 billion tonnes. The presence of these coal mines leads to air pollution.

The cities of different tiers have been chosen where air pollution is a major issue. To summarise, the cities with a large population or with a high emission rate of air pollutants affecting human health are considered. Around 600 normalized data points for each city have been used for the analysis, which is divided into train and test data in the ratio of 4 : 1. The model has been tested on the data for 100 days for each city.

### 5.2. AQI Prediction Results Using Fractional-Order Gradient Learning

**Figure 3:**
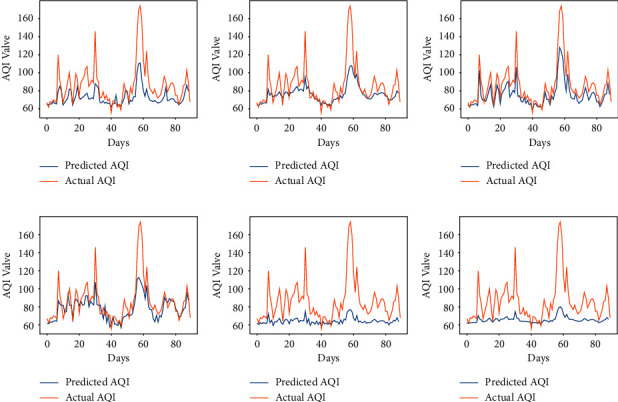
Predicted vs. actual AQI of Bengaluru city for the period of 100 days from 29-08-2019 to 07-12-2019. (a) *α* = 6/9. (b) *α* = 7/9. (c) *α* = 8/9. (d) *α* = 1. (e) *α* = 10/9. (f) *α* = 11/9.

**Figure 4:**
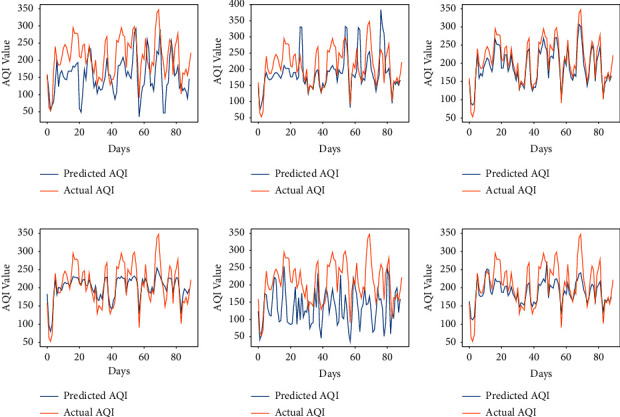
Predicted vs. actual AQI of Kolkata city for the period of 100 days from 28-10-2019 to 04-02-2020. (a) *α* = 6/9. (b) *α* = 7/9. (c) *α* = 8/9. (d) *α* = 1. (e) *α* = 10/9. (f) *α* = 11/9.

**Figure 5:**
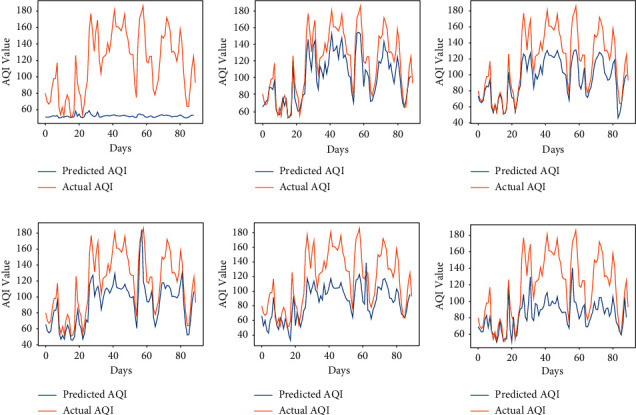
Predicted vs. actual AQI of Hyderabad city for the period of 100 days from 29-09-2019 to 06-01-2020. (a) *α* = 6/9. (b) *α* = 7/9. (c) *α* = 8/9. (d) *α* = 1. (e) *α* = 10/9. (f) *α* = 11/9.

**Figure 6:**
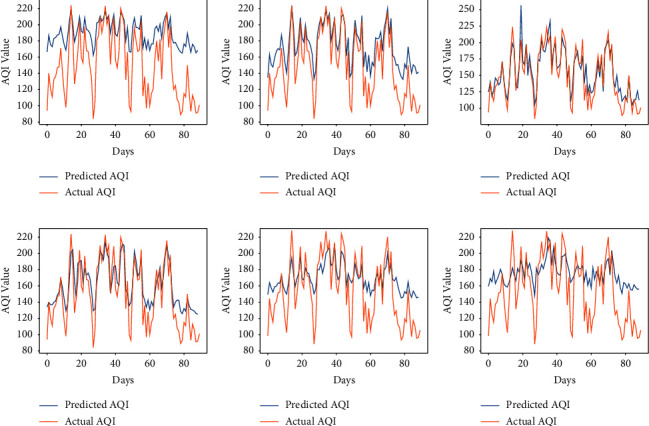
Predicted vs. actual AQI of Patna city for the period of 100 days from 26-03-2019 to 04-07-2019, (a) *α* = 6/9. (b) *α* = 7/9. (c) *α* = 8/9. (d) *α* = 1. (e) *α* = 10/9. (f) *α* = 11/9.

**Figure 7:**
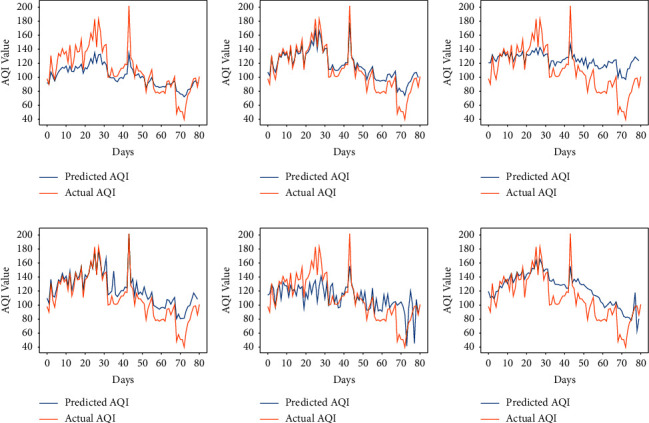
Predicted vs. actual AQI of Telchar city for the period of 100 days from 01-03-2020 to 08-6-2020. (a) *α* = 6/9. (b) *α* = 7/9. (c) *α* = 8/9. (d) *α* = 1. (e) *α* = 10/9. (f) *α* = 11/9.

The performance of the vanilla RNN in predicting AQI values of each city using the fractional backpropagation algorithm has been analyzed. To assess the performance of the model, RMSE and MAPE are computed. The prediction performance of the RNN using the fractional gradient descent algorithm with values of fractional orders in the neighborhood of 1 is compared with the performance of the RNN with the traditional integer-order gradient descent algorithm where the order remains 1. The values of fractional order *α* which are considered are 6/9, 7/9, 8/9, 1, 10/9, and 11/9 [49]. The results obtained at different orders using the proposed approach can be seen in Table 2. The graphs in Figures 3–7 show the comparison between actual and predicted output for Bengaluru, Kolkata, Hyderabad, Patna, and Talcher, respectively. In all the graphs, the expected output and the actual output are represented by the yellow lines and blue lines, respectively. It can be observed that the least RMSE and MAPE are acquired by the vanilla RNN at some fractional orders, either on 7/9 or 8/9 for all the cities. The model achieved minimum RMSE and MAPE of 13.22 and 06.11%, respectively, at *α*=8/9 for Bengaluru. Also, the minimum RMSE and MAPE are found to be 19 and 7.02% for Kolkata and 23.85 and 7.43% for Patna at *α*=8/9. Moreover, the minimum RMSE and MAPE are found to be 7.41 and 3.22% for Hyderabad and 11.40 and 4.15% for Talcher at *α*=7/9. Thus, it can be concluded from the results that the fractional-order gradient is more accurate than the integer-order gradient algorithm. Moreover, the proposed model performed best for *α*=0.7 by achieving the least MAPE of 3.22% for Hyderabad among all the cities.

### 5.3. Comparison of Results Obtained by the Proposed Method and LSTM

**Figure 8:**
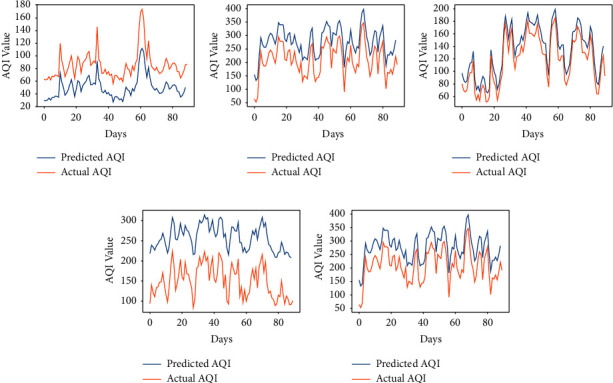
Predicted vs. actual AQI of all cities for 100 days using LSTM, (a) Bengaluru. (b) Kolkata. (c) Hyderabad. (d) Patna. (e) Telchar.

The prediction of AQI for the same set of datasets of all cities has been done using LSTM as well with the same number of timestamps, nodes, and the same procedure for inputs as done for the fractional RNN. The obtained results are also shown in Table 2. It can be concluded from the table that the performance of the LSTM is comparable to the vanilla RNN with fractional gradient learning. The RMSE values obtained by fractional gradient learning on Vanilla RNN are 23.85 and 11.40 which are lesser than 24.12 and 13.11 RMSE achieved by LSTM, corresponding to Talcher and Patna, respectively. In addition, the RMSE of the fractional-based RNN and LSTM is found to be 19 for Kolkata which is equal. Similarly, the MAPE for Hyderabad and Patna is found to be 03.22% and 7.43%, respectively, by the vanilla RNN with fractional gradient learning, whereas higher MAPE of 3.45% and 7.83% for Hyderabad and Patna, respectively, is achieved by LSTM. For Talcher, MAPE is found to be 4.15% corresponding to the fractional gradient-based RNN and is found to be 4.11% for LSTM which are almost equivalent. It can be seen that the least MAPE of 3.22% is obtained by the fractional gradient-based RNN for Hyderabad as compared to other cities and models.  Figure 8 shows the comparison between the expected AQI value and the AQI value predicted for all the cities by LSTM.

### 5.4. Prediction Results of Major Pollutants in Kolkata Using Different Fractional-Order Gradient Learning

**Figure 9:**
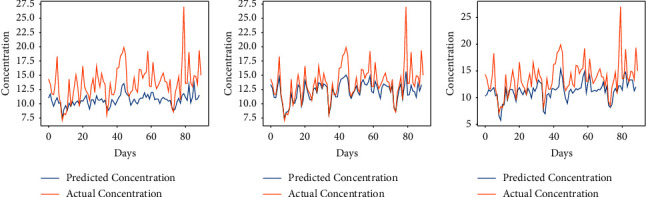
Predicted vs. actual sulphur dioxide (SO_2_) in air in Kolkata for the period of 100 days from 28-10-2019 to 04-02-2020. (a) *α* = 7/9. (b) *α* = 8/9. (c) *α* = 1.

**Figure 10:**
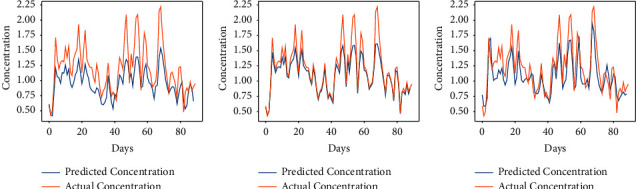
Predicted vs. actual carbon monoxide (CO) in air in Kolkata for the period of 100 days from 28-10-2019 to 04-02-2020. (a) *α* = 7/9. (b) *α* = 8/9. (c) *α* = 1.

**Figure 11:**
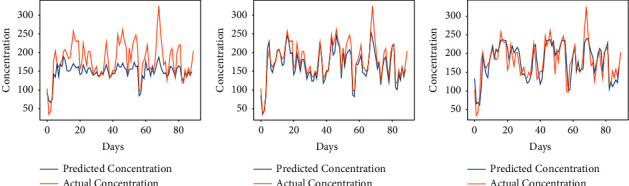
Predicted vs. actual particulate matter (PM_10_) in air in Kolkata for the period of 100 days from 28-10-2019 to 04-02-2020. (a) *α* = 7/9. (b) *α* = 8/9. (c) *α* = 1.

The proposed approach has been implemented for the prediction of the concentration of major pollutants such as SO_2_, CO, and PM_10_. Here, the considered time is also the same as used in the prediction of the AQI of Kolkata. As we have seen in the above section, the performance of the algorithm is found to be better either for *α* = 7/9 or 8/9. So, we have considered these two fractional values to compare the results with the integer-order-based learning of the vanilla RNN. Figures 9–11 show the comparison between expected air pollutant concentrations and actual concentrations in Kolkata. It can be observed from Table 3 that minimum RMSE and MAPE for each city are attained at order *α* = 8/9, thus outperforming the traditional integer-order learning for the vanilla RNN. Moreover, the least MAPE of 4.70% is achieved in the prediction of CO, and thus, the proposed model is better for predicting the concentrations of CO as compared to other pollutants.

## 6. Conclusion

In this study, the fractional-order gradient has been used in the backpropagation of the vanilla RNN for the AQI prediction of five Indian cities of all tiers. The proposed approach has been used for the prediction of major air pollutants in tier I Kolkata city. Through the results of prediction of AQI of multiple cities and prediction of air pollutants, it has been observed that the minimum error on predictions is achieved at a fractional order. Most cities achieve better results when the order is equal to 8/9. The architecture of the vanilla RNN is much simpler than the structure/functioning of an LSTM, but the predictions made by RNNs with fractional gradient-based backpropagation are comparable and sometimes even better than LSTM with the integer-order gradient descent algorithm. Achieving lesser RMSE and MAPE with simpler architecture shows the effectiveness of fractional gradient over integer-order gradient descent. The least MAPE value is found for Hyderabad by using the fractional gradient-based RNN as compared to other cities and models. In addition, the least MAPE is achieved during predictions of CO concentrations in Kolkata. Therefore, the proposed model is better in the prediction of AQI values of Hyderabad as compared to other cities and CO concentrations in the air of Kolkata as compared to other air pollutants. From the results, it can be seen that RMSE is more for Kolkata and Patna. Patna is even amongst the world's top 10 most polluted cities, and particulate matter is increasing in Kolkata each year. Hence, the memory property of fractional derivatives can be well exploited with deep neural networks for dealing with more complex and dynamic data.

## 7. Future Scope

This study can be extended by predicting other air pollutants in the cities, and from there, AQI values can also be predicted. Using this strategy, major air pollutants in a city can be detected and stringent actions can be taken accordingly to prevent further damage. A portfolio of economic activities can be created considering the air quality of the particular city and also detecting the most affecting gases among them in the future. The order of the derivative is chosen manually in this study, due to which results are evaluated only on a few values of order. Hence, there is a need to develop an adaptive method that automatically evaluates the optimal order for a particular city or a set of data. Methods like particle swarm optimization (PSO) and genetic algorithms can be employed for optimizing the order of differentiation. Fractional gradient descent can be used with suitable architectures for different cities. Through the results of predictions of various gases of the city, we can find a better way to develop in a sustainable way.

## Figures and Tables

**Table 1:** Data samples of all cities.

**Bengaluru**							
**Date**	2019-09-01	2019-09-02	2019-09-03	2019-09-04	2019-09-05	2019-19-06	2019-19-07
**AQI**	61	48	51	51	56	64	63

**Kolkata**							
**Date**	2018-06-16	2018-06-17	2018-06-18	2018-06-19	2018-06-20	2018-06-21	2018-06-22
**AQI**	119	113	107	148	94	100	60

**Hyderabad**							
**Date**	2017-11-01	2017-11-02	2017-11-03	2017-11-04	2017-11-05	2017-11-06	2017-11-07
**AQI**	150	156	158	114	91	80	94

**Patna**							
**Date**	2017-11-05	2017-11-06	2017-11-07	2017-11-08	2017-11-09	2017-11-10	2017-11-11
**AQI**	276	289	286	354	430	439	429

**Talcher**							
**Date**	2018-02-08	2018-02-09	2018-02-10	2018-02-13	2018-02-14	2018-02-15	2018-02-16
**AQI**	311	321	343	343	269	243	350

**Table 2:** Performance comparison of the fractional gradient-based RNN with the integer gradient-based RNN and LSTM.

Model	Bengaluru	Kolkata	Hyderabad	Patna	Talcher	Bengaluru	Kolkata	Hyderabad	Patna	Talcher
	RMSE					MAPE (%)				
*α*=**6/9**	21.47	31.35	25.35	44.30	14.25	14.41	18.21	16.67	14.78	06.62
*α*=**7/9**	19.36	26.12	**07.41**	34.08	**11.40**	11.32	11.58	**03.22**	10.64	**04.15**
*α*=**8/9**	**13.22**	**19.00**	09.75	**23.85**	29.64	**06.11**	0**7.02**	05.92	**07.43**	16.30
*α*=**1**	14.78	21.37	08.58	25.44	15.96	07.15	09.31	05.23	08.07	06.11
*α*=**10/9**	24.99	28.50	11.31	38.56	21.09	16.60	12.90	05.49	11.51	06.66
*α*=**11/9**	35.20	21.85	09.95	29.53	24.51	22.54	10.50	04.99	12.90	07.90
**LSTM**	**10.78**	**19.00**	**07.02**	24.12	13.11	**05.78**	**06.61**	03.45	07.83	**04.11**

**Table 3:** RMSE corresponding to the fractional gradient-based RNN for different orders in predicting concentration of different pollutants responsible for air pollution in Kolkata city.

Pollutant	RMSE			MAPE (%)		
	*α*=**7/9**	*α*=**8/9**	*α*=**1**	*α*=**7/9**	*α*=**8/9**	*α*=**1**
**S** **O**_2_	1.9	**1.62**	1.77	7.00	**5.55**	6.83
**CO**	0.20	**0.12**	0.15	5.21	**4.20**	4.89
**P** **M**_10_	36.45	**19.25**	33.75	10.95	**6.77**	11.21

## Data Availability

The AQI dataset of five cities for 2015–2020 is considered, which is publicly available at https://cpcb.nic.in, the official portal of the Central Pollution Control Board, Government of India.
